# Helping Fill That Gap: Aging in Place After Disaster Through the Lens of Home-Based Care

**DOI:** 10.1093/geroni/igaa057.799

**Published:** 2020-12-16

**Authors:** Sue Anne Bell, Lydia Krienke, Raymond DeVries, Theodore J Iwashyna

**Affiliations:** University of Michigan, Ann Arbor, Michigan, United States

## Abstract

During a disaster, home-based care is intended to continue to function using existing care delivery models. Home-based care providers (HBCP) are often the closest contact with their clients—even during a disaster, seeing them in non-traditional care settings including shelters and hotels. This closeness and commitment to clients, gives HBCP unique insights into strategies to promote aging in place. The purpose of this study was to identify the individual and community-level support needs of older adults after a disaster through the lens of home-based care. Five focus groups were conducted with HBCP (n=27) in two disaster-affected settings: 2017’s Hurricane Irma in Florida and Hurricane Harvey in Texas. Participants were identified by contacting home health agencies listed in an open-source database of agencies receiving Centers for Medicare and Medicaid Services funding. Data was manually coded using an inductive approach and themes were iteratively identified. Forty-nine codes were identified in the preliminary analysis, which were distilled into ten themes describing factors that influence care provision during and after disasters: patient autonomy/dependence, disaster-induced trauma, reluctance to evacuate, chronic disease exacerbation, unpreparedness, systemic inequality, provider preparedness actions, strong sense of community, mistrust of governmental authority, and the uniqueness of the patient and home-based care provider relationship. The perspective offered by HBCP illustrates the complexities of community-level preparedness and informal community support for chronically ill older adults surrounding disasters. Diverse groups involved in aging and disaster response can learn from strategies employed by HBCP during disasters to improve aging in place.

